# Retrospective X-ray Assessment of Wrist Joint Ossification Centers for Age Estimation in a Regional Trauma Center of North India

**DOI:** 10.7759/cureus.110907

**Published:** 2026-06-15

**Authors:** Ankit Kumar, Ashish K Singh, Manav Sharma

**Affiliations:** 1 Forensic Medicine and Toxicology, Sanjay Gandhi Postgraduate Institute of Medical Sciences, Lucknow, IND; 2 Forensic Medicine and Toxicology, Government Medical College, Haldwani, Haldwani, IND; 3 Forensic Medicine and Toxicology, Vardhman Mahavir Medical College & Safdarjung Hospital, New Delhi, IND

**Keywords:** age estimation, epiphyseal fusion, medicolegal, ossification center, unidentified body, wrist joint

## Abstract

Background

Age estimation through radiological assessment of skeletal maturation is an important component of forensic and medicolegal practices, particularly in cases involving unidentified individuals, criminal responsibility, employment disputes, and absence of authentic birth records. Ossification and epiphyseal fusion around the wrist joint are considered reliable indicators for estimating chronological age during adolescence and early adulthood. Regional and ethnic variations in skeletal maturation necessitate population-specific standards. This study aimed to retrospectively evaluate the pattern of ossification and epiphyseal fusion around the wrist joint in a Northern Indian population.

Methodology

This cross-sectional observational study was conducted in the Department of Forensic Medicine and Toxicology under Apex Trauma Centre, Sanjay Gandhi Postgraduate Institute of Medical Sciences, Lucknow, Uttar Pradesh, India. A total of 174 radiographs of the wrist joint from participants aged 14-24 years were analyzed, including 114 (65.52%) males and 60 (34.48%) females. Participants with chronic illnesses, endocrine disorders, musculoskeletal abnormalities, malnutrition, or unreliable age records were excluded. Radiological assessment included evaluation of epiphyseal fusion at the distal radius and ulna and ossification status of carpal bones. Fusion was classified as non-fusion, partial fusion, or complete fusion.

Results

Females demonstrated earlier epiphyseal fusion compared to males. At the lower end of the radius and ulna, partial fusion was observed as early as 14-15 years in 10 (62.5%) of 16 females and in 3 (20%) of 15 males at 15-16 years. The earliest complete fusion occurred at 16-17 years in 3 (33.33%) of 9 females and at 17-18 years in 3 (50%) of 6 males. Complete fusion at both the distal end of the radius and ulna was established in all participants (male or female) above 18 years of age. Ossification center for each carpal bone appeared in all participants.

Conclusions

Radiological examination of ossification centers around the wrist joint is a reliable method for age estimation in adolescents and young adults. The findings provide valuable regional reference data for the population of Eastern Uttar Pradesh and may assist forensic experts in medicolegal age determination.

## Introduction

Age estimation of an individual is a fundamental task in forensic medicine and holds significant medicolegal importance in both criminal and civil proceedings [1]. The legal framework governing age determination in India has been substantially shaped by the Supreme Court through Pratap Singh v. State of Jharkhand [2] (relevant date for determining juvenility is the date of offence), Hari Ram v. State of Rajasthan [3] (extending juvenile justice protections to all persons below 18 years of age), and Jarnail Singh v. State of Haryana [4] (establishing the hierarchy of documentary and medical evidence for age assessment).

Recent studies, supported by the Scientific Working Group for Forensic Anthropology (SWGANTH) Recommendations on Age Estimation, indicate that skeletal analysis remains a fundamental and widely accepted approach to age estimation in forensic investigations [5]. The wrist joint, comprising the lower ends of the radius and ulna along with the carpal bones, has been extensively studied for this purpose owing to its accessibility and well-defined epiphyseal activity during adolescence and early adulthood.

Several investigators have studied ossification patterns in Indian populations, including Banerjee and Agarwal [6], Nemade et al. [7], Bhise et al. [8], Patel et al. [9] Kadam and Viswanathan [10], Prasad et al. [11], Rajdev et al. [12], Vaishnawa et al. [13], Singh et al. [14], Krishnamoorthy et al. [15], Gaddewar and Meshram [16], Leena et al. et al [17], Kishore Kumar et al. [18], Biswas et al. [19], Ramesh Babu et al. [20]. However, significant regional and ethnic variations have been observed across India, necessitating population-specific studies. The Director General of Health Services Survey Committee [21] also recommended zone-wise, age-related ossification studies in India. The present study was undertaken to document ossification activity around the wrist joint in the young population of Lucknow, Uttar Pradesh, India, to generate reliable data for medicolegal age estimation in this region.

## Materials and methods

Study design and setting

This was a retrospective cross-sectional study conducted in the Department of Forensic Medicine and Toxicology and the Emergency Room, Apex Trauma Centre, Sanjay Gandhi Postgraduate Institute of Medical Sciences, Lucknow, Uttar Pradesh, India.

Study population

The study comprised 174 participants (114 males, 60 females) aged 14 to 24 years who attended the Trauma Centre during the study period of January 2025 to December 2025.

Inclusion and exclusion criteria

Participants were eligible if they were 14 to 24 years old and had a history of trauma to the wrist, hand, or forearm that required radiographic evaluation of the wrist joint. Exclusion criteria included chronic systemic illnesses affecting growth or bone development, upper-limb musculoskeletal disorders, endocrine disorders affecting growth or skeletal maturation, and evidence of severe nutritional deficiency or chronic malnutrition. Participants were also excluded if age could not be reliably determined from records or if wrist injuries or fractures obscured the epiphyseal region, preventing accurate radiological assessment.

Ethical considerations

Ethical approval was obtained from the Ethics Committee of Sanjay Gandhi Postgraduate Institute of Medical Sciences, Lucknow (IEC code: 2026-79-IP-EXP-68). No personally identifiable patient data were collected. All data were anonymized and stored on a password-protected device.

Data collection

A structured data collection sheet was prepared for the present study to collect demographic details and relevant clinical information from case files. All X-ray films were reviewed on an X-ray viewing box and a computer. Radiological findings about epiphyseal activities at the wrist joint, including the lower end of the radius, the lower end of the ulna, and the carpal bones, were documented in the data collection sheet.

Statistical analysis

Data from the collection sheets were entered into Microsoft Excel and then analyzed using SPSS Statistics for Windows, version 26.0 (IBM Corp., Armonk, NY, USA). Categorical variables, including age group, sex, and epiphyseal fusion status at the distal radius and ulna (non-fusion, partial fusion, complete fusion), are summarized as frequencies and percentages. Cross-tabulations are used to present the distribution of participants by age and sex and the distribution of epiphyseal fusion stages at the distal radius and ulna across age groups and sex. As the study focused on describing the pattern and timing of epiphyseal fusion rather than testing specific hypotheses, no inferential statistical tests were performed, and the results are presented as descriptive statistics.

Classification of epiphyseal activity

Epiphyseal status at each site was classified as follows: (i) non-fusion (NF), where the epiphysis is not fused with the diaphysis; (ii) partial fusion (PF), indicating partial bridging of the epiphyseal plate; and (iii) complete fusion (CF), representing complete obliteration of the epiphyseal plate [22,23].

## Results

Demographic distribution

A total of 174 participants were included in the wrist ossification study. Among these, 114 (65.51%) were male, and 60 (34.48%) were female. The age-wise and sex-wise distribution of participants is presented in Table 1.

**Table 1:** Distribution of participants by age group and sex.

Age group (years)	Male, n (%)	Female, n (%)	Total
14–15	18 (10.34%)	16 (9.20%)	34 (19.54%)
15–16	15 (8.62%)	14 (8.05%)	29 (16.67%)
16–17	18 (10.34%)	9 (5.17%)	27 (15.52%)
17–18	6 (3.45%)	5 (2.87%)	11 (6.32%)
18–19	7 (4.02%)	3 (1.72%)	10 (5.75%)
19–20	5 (2.87%)	4 (2.30%)	9 (5.17%)
20–21	9 (5.17%)	2 (1.15%)	11 (6.32%)
21–22	10 (5.75%)	3 (1.72%)	13 (7.47%)
22–23	14 (8.05%)	3 (1.72%)	17 (9.77%)
23–24	12 (6.90%)	1 (0.57%)	13 (7.47%)
Total	114 (65.52%)	60 (34.48%)	174 (100%)

Fusion of epiphysis at the lower end of radius

The age-wise distribution of epiphyseal fusion status at the lower end of the radius is presented in Table 2. Complete union of the lower end of the radius bone was seen in five (100%) of five cases in females at 17-18 years and seven (100%) of seven males at 18-19 years.

**Table 2:** Age-wise distribution of participants according to fusion of epiphysis at the lower end of the radius. NF = non-fusion; PF = partial fusion; CF = complete fusion

Age group (years)	Male NF, n (%)	Male PF, n (%)	Male CF, n (%)	Female NF, n (%)	Female PF, n (%)	Female CF, n (%)
14–15	18 (100%)	0	0	6 (37.5%)	10 (62.5%)	0
15–16	12 (80%)	3 (20%)	0	4 (28.5%)	10 (71.5%)	0
16–17	3 (16.6%)	15 (83.4%)	0	0	6 (66.7%)	3 (33.3%)
17–18	0	3 (50%)	3 (50%)	0	0	5 (100%)
18–19	0	0	7 (100%)	0	0	3 (100%)
19–20	0	0	5 (100%)	0	0	4 (100%)
20–21	0	0	9 (100%)	0	0	2 (100%)
21–22	0	0	10 (100%)	0	0	3 (100%)
22–23	0	0	14 (100%)	0	0	3 (100%)
23–24	0	0	12 (100%)	0	0	1 (100%)

Fusion of epiphysis at the lower end of ulna

The age-wise distribution of epiphyseal fusion status at the lower end of the ulna is presented in Table 3. Complete union of the lower end of the ulna bone was seen in five (100%) of five cases in females at 17-18 years and seven (100%) of seven males at 18-19 years.

**Table 3:** Age-wise distribution of participants according to fusion of epiphysis at the lower end of the ulna. NF = non-fusion; PF = partial fusion; CF = complete fusion

Age group (years)	Male NF, n (%)	Male PF, n (%)	Male CF, n (%)	Female NF, n (%)	Female PF, n (%)	Female CF, n (%)
14–15	18 (100%)	0	0	6 (37.5%)	10 (62.5%)	0
15–16	12 (80%)	3 (20%)	0	4 (28.5%)	10 (71.5%)	0
16–17	3 (16.6%)	15 (83.4%)	0	0	6 (66.7%)	3 (33.3%)
17–18	0	3 (50%)	3 (50%)	0	0	5 (100%)
18–19	0	0	7 (100%)	0	0	3 (100%)
19–20	0	0	5 (100%)	0	0	4 (100%)
20–21	0	0	9 (100%)	0	0	2 (100%)
21–22	0	0	10 (100%)	0	0	3 (100%)
22–23	0	0	14 (100%)	0	0	3 (100%)
23–24	0	0	12 (100%)	0	0	1 (100%)

Carpal bone ossification

As the study population was aged 14 years and above, ossification centers for all carpal bones had appeared.

Age group-wise observations

14-15 Years

This age group comprised 34 (19.54%) participants: 18 (10.34%) males and 16 (9.20%) females. At the lower end of the radius and ulna, all males (n = 18, 100%) demonstrated non-fusion of the epiphysis. Among females, 6 of 16 (37.5%) showed non-fusion, while the remaining 10 of 16 (62.5%) showed partial fusion.

15-16 Years

This age group comprised 29 (16.67%) participants: 15 (8.62%) males and 14 (8.05%) females. At the lower end of the radius and ulna, 12 of 15 males (80%) had non-fusion, and 3 of 15 (20%) had partial fusion. Among females, 4 of 14 (28.57%) had non-fusion, and 10 of 14 (71.43%) had partial fusion.

16-17 Years

This age group comprised 27 (15.52%) participants: 18 (10.34%) males and 9 (5.17%) females. At the lower end of the radius and ulna, 3 of 18 males (16.6%) had non-fusion, and 15 of 18 (83.33%) had partial fusion. Among females, 6 of 9 (66.66%) had partial fusion, and 3 of 9 (33.33%) showed complete fusion, representing the earliest age of complete fusion observed in females.

17-18 Years

This age group comprised 11 (6.32%) participants: six (3.54%) males and five (2.87%) females. At the lower end of the radius and ulna, three of six males (50%) had partial fusion, and three of six (50%) had complete fusion, representing the earliest age of complete fusion observed in males. All five (100%) females had complete fusion.

18-19 Years

This age group comprised 10 (5.15%) participants: seven (4.02%) males and three (1.72%) females. At the lower end of the radius and ulna, all males (n = 7, 100%) had complete fusion, and all females (n = 3, 100%) demonstrated complete fusion.

19-20 Years

This age group comprised nine (5.17%) participants: five (2.87%) males and four (2.30%) males. All males (n = 5, 100%) and all females (n = 4, 100%) exhibited complete fusion at both the lower end of the radius and the lower end of the ulna.

20-24 Years

In all subsequent age groups (20-21, 21-22, 22-23, and 23-24 years), all male and female participants demonstrated complete epiphyseal fusion at the lower end of the radius and ulna.

## Discussion

In the present study, non-fusion of the epiphysis at the lower end of the radius was the predominant finding among males in the 14-15- and 15-16-year age groups. In females of the same age groups, partial fusion was the predominant finding. The earliest complete fusion appeared at 16-17 years in three (33.33%) of nine females and at 17-18 years in three (50%) of six males. Complete fusion in both sexes was observed above 18 years of age. These findings are consistent with those of Banerjee and Agarwal [6], Patel et al. [9], Prasad et al. [11], Kishore Kumar et al. [18], and Ramesh Babu et al. [20]. Hepworth [24] reported fusion at 16-17 years.

The pattern of epiphyseal fusion at the lower end of the ulna was identical to that observed at the lower end of the radius. Non-fusion predominated among males aged 14-16 years, while partial fusion predominated in females of the same age groups. The earliest complete fusion appeared at 16-17 years in three (33.33%) of nine females and at 17-18 years in three (50%) of six males. Complete fusion in both sexes was observed above 18 years of age. These findings are consistent with observations by Banerjee and Agarwal et al. [6], Vaishnawa et al. [13], Rajdev et al. [12], Ramesh Babu et al. [20], and Leena et al. [17]. Earlier fusion was also reported by Hepworth [24].

In our study, as the age group was 14-24 years, all the carpal bones had appeared. The order of appearance of carpal bones as observed in other studies was capitate, hamate, triquetral, lunate, trapezium, trapezoid, scaphoid, and pisiform [25,26].

Globally, the age ranges for distal forearm fusion observed in this study are broadly comparable to those reported in the African cohort [27]. Differences may result from genetic background, environmental exposures, nutritional status, and secular trends in growth and maturation. From a medicolegal perspective, these findings highlight the need to use locally validated skeletal age standards rather than applying foreign reference data directly when assessing age in the Indian population.

Our findings demonstrate that females achieve epiphyseal fusion earlier than males, with complete fusion of the distal radius and ulna occurring approximately one year earlier in females. This difference is consistent with previous Indian studies on wrist and long-bone ossification [6-9,12,13,15,17], highlighting the necessity for sex-specific reference standards in medicolegal age estimation.

Our study found that all females had complete fusion of the distal radius and ulna by 17 to 18 years, and all males by 18 to 19 years. These results are useful for determining whether someone has reached the age of majority in India, especially when documents are missing or are in dispute. Using wrist X-rays and local standards from this research can help forensic experts estimate age more accurately and make age determination more objective in legal cases. The study also provides region-specific data that match the recommendations of the Director General of Health Services Survey Committee [21] for zone-wise ossification studies in India.

For forensic purposes, the findings indicate that complete fusion of both the distal radius and ulna strongly supports an age above 17 years in females and 18 years in males. In contrast, the presence of non-fusion, particularly in males, is more consistent with an age of less than 14 years. Integrating these wrist-based observations with additional skeletal and dental indicators, as well as documentary evidence in accordance with Supreme Court guidelines [2-4], can enhance the robustness, transparency, and defensibility of age determinations in legal proceedings.

Some limitations should be considered when interpreting these findings. The sample consisted exclusively of hospital attendees, which may reduce its representativeness of the general population. Inclusion was limited to individuals undergoing clinically or medicolegally indicated radiographic examinations. This resulted in an unequal distribution of participants across age groups. The small number of participants in certain age categories may have limited the assessment of variability in ossification patterns and developmental progression. Age assignment relied in part on government-issued records, such as birth certificates and Aadhaar documents, whose authenticity and accuracy could not be independently verified in all cases. Potential confounding factors, including nutritional status, lifestyle, socioeconomic conditions, climatic influences, and intercurrent illnesses, were not systematically evaluated and may have influenced skeletal maturation. Some occult physeal injuries, especially Salter-Harris type I fractures, may not have been detectable on plain radiographs, even though cases with evident growth plate involvement were excluded. This limitation introduces a potential confounding factor in evaluating epiphyseal fusion. Substantial inter-regional and population-specific variation in the timing of ossification and epiphyseal fusion has been documented. Therefore, these findings should be applied cautiously and not generalized to other populations without further validation.

## Conclusions

Radiographic evaluation of ossification centers around the wrist joint is a practical and reliable approach for estimating age in adolescents and young adults in this population. In a cohort from a regional trauma center in Eastern Uttar Pradesh, females consistently demonstrated earlier epiphyseal fusion of the distal radius and ulna than males. By late adolescence, complete fusion at both sites was observed in all participants. These population-specific findings provide valuable reference ranges for forensic practitioners estimating age in the absence of reliable documentary evidence. Although the study primarily addressed forensic age estimation, the findings may also be relevant for clinical bone age assessment among children. However, the generalizability of these findings to metabolic and endocrine evaluations in children requires further validation through prospective studies across diverse Indian populations. The integration of regionally derived standards into routine medicolegal practice is expected to enhance the accuracy, reproducibility, and defensibility of skeletal age estimation among young individuals.
